# Effect of Zingiber Cassumunar Roxb. Phonophoresis Versus Aqua Sonic Gel on Pain, Range of Motion, and Functional Disability in Patients With Osteoarthritis of Knee: A Randomized Controlled Trial

**DOI:** 10.7759/cureus.32760

**Published:** 2022-12-20

**Authors:** Medhavi V Joshi, Pratik Phansopkar

**Affiliations:** 1 Musculoskeletal Physiotherapy, Ravi Nair Physiotherapy College, Datta Meghe Institute of Medical Sciences, Wardha, IND

**Keywords:** randomized controlled study, knee osteoarthritis, physiotherapy intervention, physiotherapy, otago exercise, phonophoresis

## Abstract

The aim of this trial was to collate, analyze, and compare the effectiveness of phonophoresis and ultrasound adjunct to Otago exercise program for pain, knee range of motion, and functional disability in patients with grades I and II of knee osteoarthritis. This is a single-blind randomized control study. A total of 52 patients with pre-diagnosed osteoarthritis on radiological investigation were included in the study. They were then randomly assigned into two groups: group A (n=26) received the experimental treatment, which included phonophoresis, and group B (n=26) subjects were treated with conventional physiotherapy. The interventions were given for six days/week for two weeks. Pain via the visual analog scale, knee range of motion via a goniometer, level of functional disability through the Western Ontario and McMaster Universities Osteoarthritis Index, and balance through the star excursion balance test were evaluated. Measurements of the outcome were taken prior to initiation of intervention and on the day of the last session, succeeding the treatment. Statistically, both the groups showed significant improvement in pain, range, dynamic balance, and functioning. Between the two groups, no clinically significant difference was present for balance, but the intergroup comparison for pain and functions was statistically and therefore clinically significant. Phonophoresis treatment was observed to be effective in treating pain in osteoarthritis. Combined Otago exercises and phonophoresis with modified gel can be used to achieve superior clinical results.

## Introduction

Globally, a painful knee in the elderly population is a major disabling condition. Pain in such older individuals is predominantly associated with osteoarthritis (OA) of the knee. A total of 303 million people were affected globally in 2017, with a prevalence of 16.0% and 22.9% in a population aged above 15 and 40 years, respectively [1,2]. Knee OA, a rheumatic musculoskeletal disorder, is a condition that affects both the tibiofemoral and patellofemoral compartments of the knee. Differences in lifestyle such as sitting habits, toileting habits, level of involvement in sporting activities, and associated injuries are considered to be pre-deciding factors of which compartment will initially be affected [3]. The pathophysiological process begins with altered mechanical loading of bone, cartilage, and ligament. This leads to proteolytic destruction of the cartilage matrix by metalloproteinase and chondrocyte death [4]. Post-cartilage destruction, the subchondral bone is subjected to the formation of sclerosis, thickening, and osteophytosis. A simultaneous process of inflammation of synovial fluid occurs, and the inflamed synoviocytes then produce inflammatory cytokines that act to facilitate the production of the negative effect of metalloproteinase [5,6]. Chronic synovial inflammation precipitates into central and peripheral centralization and nociceptive activation. An amalgamation of all the above processes leads to stiffness, reduced exercise, weakness due to primary impairment pain, and, consequently, joint laxity and reduced proprioception prevail. There has been an increase in the number of people falling in the category of borderline obese and above, which is a major modifiable risk factor predisposing an individual to arthritis. According to the World Health Organization (WHO), since 1975, obesity has nearly tripled; in 2016, 1.9 billion adults were overweight (body mass index of 25 and above), and 650 of these were obese (body mass index of 30 and above). In 2016, 340 million children and adolescents aged 5-19 years were either overweight or obese. The latest statistics for 2020 show that a total of 39 million children below the age of 5 are overweight. The following statistical analysis from WHO is in parallel with the ever-increasing incidence of people affected with knee arthritis and undergoing total knee arthroplasty [7].

Physical therapy treatment involves the use of electrotherapeutic modalities to work on both reducing pain and gaining strength. Therapeutic ultrasound, a deep heating modality, produces a heating effect via mechanical sound waves, up to a minimum depth of 5 centimeters below the surface of the skin. The coupling medium is a part of the ultrasound unit essential for the transfer of electrical current as it reduced the diversion of the waves away from the tissues by minimizing air between the transducer head and skin. It is in the form of water, oil, and gels having their impedance [8,9]. Numerous research studies have included active compounds such as diclofenac gel and ibuprofen, providing a dual benefit of the coupling agent [10]. Ayurvedic herbs especially those with anti-inflammatory properties have also been used in the treatment of OA both orally and with their topical application [11]. A study shows that only 19% of individuals taking Ayurvedic treatment for OA knee took pain medicine compared to 81% of individuals taking conventional treatment therapy for the same [12]. The orally taken Ayurvedic medicines have also been shown to be effective but have few negative effects such as the color, appearance, and taste not necessarily attractive to patients [13]. Zingiber Cassumunar Roxb., with the scientific name being ginger, is an herbaceous plant. It is a traditional plant-based food spice and is used as a herbal medicine in south Asian regions specifically in northeast India [14]. Topically administrated ginger, for instance, ginger compresses, has been utilized for improving blood circulation and body fluid circulation in areas that are affected by pain, inflammation, and stiffness [15]. Studies have also demonstrated that the application of a cotton cloth soaked in hot ginger infusion, also known as ginger compress pack (GKC), over the kidney has been effective in treating inflammatory conditions such as arthritis, bronchitis, and some forms of depression as well [16]. The findings of the study revealed that during the period of application of these GKCs, the patient experienced prolonged concentrated warmth releasing the body’s tension. The properties of ginger allow for heat stimulation, anti-inflammation, and analgesia, allowing treated individuals to have a short-term detachment from pain and stress [17]. The most recent study conducted by Decha et al. used Phyllanthus amarus, a medicinal herb, mixed in a 4:11 ratio with the conventional coupling agent, for evaluating its effect on knee pain in arthritic patients [18]. We, therefore, conducted this study to find the effectiveness of topically applied ginger infusion through a process called phonophoresis.

## Materials and methods

Study design

This study was performed during the years 2021 and 2022, after approval from Institutional Ethics Committee (IEC) with IEC No. 242 and Clinical Trial Registration of India (CTRI) No. Ref No. 2021/04/043216. A research protocol of the study was published in the Journal of Pharmaceutical Research International prior to beginning of the study. Patients were thoroughly evaluated, and those (n = 52) who fulfilled the inclusion criteria were then included in the study for a duration of two weeks. After randomization with a lottery method, they were allocated into two groups: group A (n=26) and group B (n=26).

Participants

All participants were educated about the details of the intervention, research, and data confidentiality prior to the start of the study. Patients in the study were referred from either orthopedicians, tertiary health care providers, or self-referred subjects. Subjects were pre-diagnosed cases aged between 40 and 60 years with grade I or II OA on Kellgren and Lawrence scale with unilateral OA of the knees and less than 30 minutes of morning stiffness in the knees. Patients with superficial and deep sensory impairments, those who underwent total hip joint replacement, those with clinically diagnosed neurological disorders such as stroke and parkinsonism, those with current participation in another OA intervention study, those with physical disability (unable to walk even with a walking aid), and those who are unable to comprehend were excluded from the study.

Treatment procedure and intervention design

The medicinal coupling medium was made with the assistance of the Head of Department and Faculty of Ayurveda College. A small sample of 40 mL was made with trial and error method using wet ginger carbapol for achieving gel-like consistency, and a preservative. After evaluating the consistency and safety of the gel, a larger batch of 350 mL was made with the same ratio. Ultrasound is applied to the affected knee for a total of 10 sessions for two weeks, that is, five sessions/week, using a device with a 1-MHz probe for a period of 8 minutes at an intensity of 0.8 W/cm^2^ [19]. Ultrasound using Zingiber cassumunar Roxb. gel as a coupling medium is given to the experimental group A. Ultrasound with routine aqua sonic gel is given to group B. This intervention is then followed by the Otago exercise program. Alternate days balance retraining and strengthening exercises were also performed. The outcome measures were assessed at baseline prior to the intervention and after two weeks of intervention. Primary outcome measures were visual analogue scale, goniometer, and Western Ontario and McMaster Universities Osteoarthritis Index (WOMAC), while the secondary outcome measure was the star excursion balance test for dynamic balance.

## Results

Statistical analysis was conducted using descriptive and inferential statistics using the chi-square test and Student’s paired and unpaired t-test, and software used in the analysis were SPSS Version 27 (IBM Corp., Armonk, NY) and GraphPad Prism 7.0 version. A p-value of <0.05 was considered as level of significance. There was no significant difference within the two groups with respect to age, gender, and grade of OA. The comparison of means within the group and between two groups is given in the following.

Assessment of disease-specific measure of symptoms

Visual Analog Scale

Table 1 shows the mean values of pain taken on visual analog scale.

**Table 1:** Mean values of pain taken on visual analog scale The pre-test average values in the experimental and control groups were 7.73±1.05 and 7.92±0.88 respectively. After two weeks of treatment, values were 2.55±0.89 and 3.34±0.90 with their respective standard deviations. Students paired t-test was used to find the within the same group pre- and post-significance. For both the groups, pre- and post-comparison was found to be significant with t-values of 38.79 and 35.92. Independent t-test was used to compare intergroup values. The t-value is 3.3 and p-value is 0.0018, which is significant. S, significant

Group	Pre-test	Post-test	Mean difference	Student’s Paired t-test t-value
Experimental group	7.73±1.05	2.55±0.89	5.18±0.65	38.79; p=0.0001, S
Control group	7.92±0.88	3.34±0.90	4.57±0.65	35.92; p=0.0001, S
Comparison of mean difference in two groups (Student’s unpaired t-test)→			t-value	p-value
			3.3	0.0018, S

Western Ontario and McMaster Universities Osteoarthritis Index

Table 2 shows the comparison of WOMAC score statistically in two groups at pre- and post-treatment.

**Table 2:** Comparison of WOMAC score statistically in two groups at pre- and post-treatment The mean WOMAC scores pre-treatment were 63.29 and 62.25 with a standard deviation of 11.34 and 9.03 in experimental and control groups, respectively. The post-treatment values were 34.80±8.10 and 38.46±7.72, indicating a mean difference of 28.49 and 23.79, respectively. Using Student’s unpaired t-test a comparison of mean differences gave a p-value of 0.024, indicating a clinically significant difference in the WOMAC scoring of experimental and control groups. S, significant; WOMAC, Western Ontario and McMaster Universities Osteoarthritis Index

Group	Pre-test	Post-test	Mean difference	Student’s paired t-test t-value
Experimental group	63.29±11.34	34.80±8.10	28.49±6.45	21.61; p=0.0001, S
Control group	62.25±9.03	38.46±7.72	23.79±7.71	15.72; p=0.0001, S
Comparison of mean difference in two groups (Student’s unpaired t-test)→			t-value	p-value
			2.32	0.024, S

Range of motion

Statistics show that post-treatment a minimum change of 5-8 degrees was seen in after two weeks of intervention in both the groups.

Measure of balance

Star excursion balance test: affected extremity

**Figure 1:**
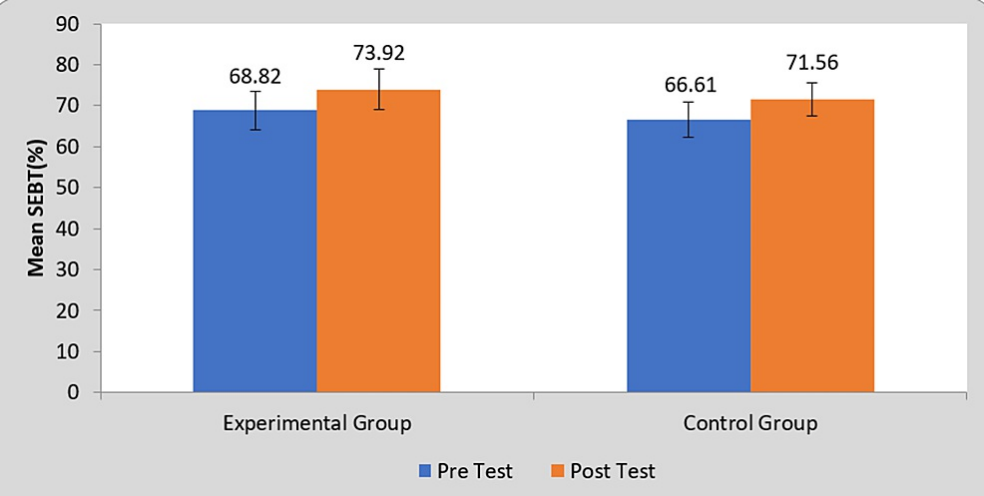
Bar graph representing the mean values in percentage for star excursion balance test for both the groups

Figure 1 represents the average value of summated values for both the groups for star excursion balance test of the affected extremity. The pre-test values were 68.82 and 66.61 with a standard deviation of 4.68 and 4.32 for the experimental and control groups, respectively. The post-test values with standard deviation were 73.92±4.99 and 71.56±4.13. The intergroup comparison with Student’s unpaired t-test showed no statistically significant difference after two weeks of intervention with the Otago exercise program.

## Discussion

In this study, individuals with grades I or II of OA who had appreciable levels of pain during the pre-treatment phase in both groups were present. The causative factor for dependent lifestyle, reduced physical activity, and an increased apprehension in both the groups was pain. These findings are parallel with previous studies that indicate reduced activity levels in affected individuals [20-22]. Similarly, a study suggests that symptoms of OA contribute to functional limitations [23]. Before the administration of intervention, clinical features were standardized, and therefore no significant difference was observed in their characteristic. We aimed to determine the added effect of phonophoresis, peculiarly with zingiber gel on knee pain and inflammation in patients with OA. To our knowledge, previous studies have not combined this particular coupling medium for phonophoresis and Otago exercise rehabilitation program as an intervention strategy in this population. To improve the transdermal drug transmission, the technique of phonophoresis was developed where a medicinal ointment was used with the coupling medium of ultrasound for its percutaneous absorption through the stratum corneum of the skin [24,25]. We could confirm the hypothesis that the medicinal gel phonophoresis has benefits mildly superior to those with conventional ultrasound therapy in this subgroup of the targeted population as the primary outcome measures illustrated statistically significant improvements in pain and performance of functional activities. With thermal and non-thermal benefits of ultrasound, the inherent anti-inflammatory property of ginger used in the gel adds to the therapeutic benefit of intervention. Comparison of pain, measured on a visual analog scale having a reliability and validity of 0.94 and 0.97, was done using an unpaired t-test. The calculated values for intergroup comparison showed a t-value of 3.3. In this study, a significant effect on pain was observed, as a change of even 30 mm in the severity of pain is representative of minimum clinically improved difference (MCID) [26,27]. Results also demonstrate a significant improvement in the functioning of the individuals after 10 sessions of intervention with both therapeutic ultrasound and exercise rehabilitation program. The positive findings of the study corroborate with the results of other research, where a therapeutic duration of 8 minutes of ultrasound had a significant improvement in WOMAC functional scores [28].

The study also includes the Otago exercise program as a part of the intervention given to both the experimental and control group. It also states that overall physical activity is at suboptimal levels regardless of pain severity in OA patients and therefore emphasis should be made to include exercise programs in rehabilitation strategies and daily activities [29]. We have utilized the exercises of this home-based Otago exercise program to be administrated in a healthcare setting under the observation of a therapist. The strengthening and balance exercises are given on alternate days for two weeks. A study by Cederbom and Arkkukangas on community-dwelling elderly showed that Otago exercise program had a significant effect on pain reduction. Their results showed exercises done two-three times a week were also effective for pain management [30]. This finding when correlated with our study is suggestive that the reduction in pain can also be attributed to the regularly performed strengthening and balance re-training exercises. As the duration of our intervention is short, a long-term follow-up would give a piece of stronger evidence for the use of supervised Otago exercise program as an adjunct for pain reduction management in terms of non-pharmacological treatment. These exercises in previously studied articles have been associated with having an impact on fall prevention in elderly individuals at high risk [31]. As from the previous studies, we know that Otago exercise program works on fall prevention by improving strength, flexibility, and balance; in this study, we therefore utilized a dynamic balance test, which is the star excursion balance test, which is a functional performance-based test. It is an indirect assessment of their strength and range. The outcome measures used in previous studies are time up and go (TUG), 30-second chair test, four-stage balance test, step test, modified falls efficacy scale, and gait velocity [31]. As our study did not record BMI values at any time during intervention, statistical evidence for the above finding will require further research. Further similar studies can be conducted including individuals with bilateral knee OA or with grades III and IV of knee OA, or a targeted population can be taken, e.g., people with obesity, farmers, or individuals with previous ligament injury. Previous literature shows that pain management has to be combined with a set of exercises for the most beneficial outcome in treating OA conservatively.

## Conclusions

We conclude that application of phonophoresis using wet ginger nano gel along with strengthening and balance retraining exercises performed daily are more effective in relieving pain and improving functional status in patients with grades I and II of OA compared to conventional therapeutic ultrasound. It is a cost-effective, safe treatment that can be used routinely for treatment of knee pain and other conditions with pain arising from degenerative changes or pain associated with inflammatory conditions, having benefits of natural products. This method of phonophoresis will also increase the usage of natural products and their properties in treating pain.
